# Downregulation of lncRNA *PpL-T31511* and *Pp-miRn182* Promotes Hydrogen Cyanamide-Induced Endodormancy Release through the PP2C-H_2_O_2_ Pathway in Pear (*Pyrus pyrifolia*)

**DOI:** 10.3390/ijms222111842

**Published:** 2021-10-31

**Authors:** Liang Li, Jinhang Liu, Qin Liang, Yu Feng, Chao Wang, Shaohua Wu, Yongyu Li

**Affiliations:** 1 College of Horticulture, Fujian Agriculture and Forestry University, Cangshan District, Fuzhou 350002, China; 2160305002@fafu.edu.cn (L.L.); 1170371002@fafu.edu.cn (J.L.); 1180305007@fafu.edu.cn (Q.L.); 1190305004@fafu.edu.cn (Y.F.); 3200330049@fafu.edu.cn (C.W.); wushaohua@fafu.edu.cn (S.W.); 2 Fruit Research Institute, Fujian Academy of Agricultural Sciences, Jinan District, Fuzhou 350013, China

**Keywords:** *Pyrus pyrifolia*, endodormancy, hydrogen cyanamide, PP2Cs, LncRNA *PpL-T31511*, *Pp-miRn182*, H_2_O_2_

## Abstract

Bud endodormancy is an important, complex process subject to both genetic and epigenetic control, the mechanism of which is still unclear. The endogenous hormone abscisic acid (ABA) and its signaling pathway play important roles in the endodormancy process, in which the type 2C protein phosphatases (PP2Cs) is key to the ABA signal pathway. Due to its excellent effect on endodormancy release, hydrogen cyanamide (HC) treatment is considered an effective measure to study the mechanism of endodormancy release. In this study, RNA-Seq analysis was conducted on endodormant floral buds of pear (*Pyrus pyrifolia*) with HC treatment, and the HC-induced PP2C gene *PpPP2C1* was identified. Next, software prediction, expression tests and transient assays revealed that lncRNA *PpL-T31511*-derived *Pp-miRn182* targets *PpPP2C1*. The expression analysis showed that HC treatment upregulated the expression of *PpPP2C1* and downregulated the expression of *PpL-T31511* and *Pp-miRn182*. Moreover, HC treatment inhibited the accumulation of ABA signaling pathway-related genes and hydrogen peroxide (H_2_O_2_). Furthermore, overexpression of *Pp-miRn182* reduced the inhibitory effect of *PpPP2C1* on the H_2_O_2_ content. In summary, our study suggests that downregulation of *PpL-T31511*-derived *Pp-miRn182* promotes HC-induced endodormancy release in pear plants through the PP2C-H_2_O_2_ pathway.

## 1. Introduction

To cope with cyclic periods of environmental stress such as cold winters, perennial deciduous trees developed a strategy of going dormant during evolution [1]. Based on different stress contexts, plant dormancy was classified into three categories: paradormancy, endodormancy and ecodormancy [2]. Paradormancy occurs only in lateral buds after elongation has stopped, which is imposed by apical dominance. In autumn, perennial deciduous trees shed leaves, and the buds enter endodormancy, which is induced by cold temperatures, short photoperiods or both [3,4]. Within the family Rosaceae, the endodormancy of pear buds is induced by low temperatures [5]. Under natural conditions, the release of buds from endodormancy is a temperature-dependent process, where a genetically determined amount of accumulative chilling is required to break endodormancy [6]. The endodormant buds cannot break endodormancy under insufficient chilling accumulation during winter, which leads to unsuccessful flowering and reduced fruit production [7]. When this chilling requirement is met, pear buds shift to ecodormancy. In ecodormancy, pear buds are still repressed due to temporary environmental stresses, but this state is released once the stressful conditions cease [3]. Due to global abnormal weather, unstable chilling accumulation has seriously affected endodormancy establishment and release in many perennial deciduous trees, including pear trees, resulting in production instability and economic losses. Therefore, identifying the molecular mechanism regulating endodormancy may provide the basis for solving this key problem in agriculture. 

Bud endodormancy in winter is of economic importance and widely studied. As an effective chemical agent that could replace chilling accumulation and rapidly release buds from endodormancy, 1.0% *w*/*v* hydrogen cyanamide (HC) has been widely applied to many deciduous fruit trees such as grape (*Vitis vinifera* L.) [8], blueberry (*Vaccinium* spp.) [9], peach (*Prunus persica*) [10] and pear (*Pyrus pyrifolia*) [11]. Additionally, HC treatment combined with transcriptome analysis has been used as an effective method to reveal the mechanism of bud endodormancy [12,13]. Phytohormones are physiological factors that have been associated with bud growth and development. Metabolic pathways involved in endodormancy induction associated with abscisic acid (ABA), ethylene and auxin were identified by transcriptome analysis [14,15]. Gibberellic acid (GA), cytokinin, jasmonic acid (JA) and brassinosteroid (BR) also participated in endodormancy [15]. Among then, ABA has proved to be a crucial promoter involved in bud endodormancy [16]. It was reported that the application of ABA inhibits endodormancy release and attenuates the advancing effect of HC in grape buds [17]. HC treatment also decreased ABA levels, which coincided with the expression trends of the genes involved in the ABA metabolism and signaling pathways, according to transcriptome analysis [13,18]. *Nine-cis-epoxycarotenoid dioxygenase* (*NCED*) is a rate-limiting gene for ABA biosynthesis. The cytochrome P450 *CYP707A* family is thought to be the key regulator in ABA metabolization. *NCED* was upregulated prior to the onset of endodormancy along with an increase in ABA levels. With endodormancy release, the expression of *CYP707A* was upregulated and the levels of ABA content and *NCED* expression were decreased [11,19]. The *dormancy-associated MADS-box* gene (*DAM*), a key factor involved in dormancy regulation in Rosaceae species [7,20,21], has been reported to activate *NCED* expression by binding to the CArG motif in the *NCED* promoter [11]. As major components of the ABA signaling mechanism, type 2C protein phosphatases (PP2Cs) negatively regulate the ABA signaling pathway [22]. PP2Cs involved in seed dormancy have been reported [23]. Both PYL/RCAR ABA receptors [24] and protein DELAY OF GERMINATION1 (DOG1) [25] could form a complex with PP2Cs that inhibits the activities of PP2Cs, resulting in seed dormancy in *Arabidopsis thaliana*. Ectopic expression of wheat *PP2C-a10* in *Arabidopsis* promoted seed germination [26]. In pear buds, the levels of expression of *PpPP2Cs* decreased in endodormancy and then increased in endodormancy release. *PpPP2C-12* expression was inhibited in ABA-treated buds [18]. One week after HC treatment, *PpPP2C* was upregulated and reached the highest levels compared with water treatment [11]. Those results suggested that the upregulation of *PP2C* was associated with endodormancy release. 

In addition, energy metabolism was considered to be a key regulatory mechanism involved in bud endodormancy release [8]. Oxidation and respiratory metabolism, which provide energy for metabolic activities, were suggested to be involved in the release of floral buds from endodormancy [27]. Under HC treatment, respiration intensity suddenly decreased and then increased significantly one day later in dormant peach floral buds [28]. In addition, metabolites related to the tricarboxylic acid cycle (TCA) [9] and pentose phosphate pathway (PPP) [28] were induced by HC treatment. Oxidation and respiratory stresses trigger the production of reactive oxygen species (ROS), including hydrogen peroxide (H_2_O_2_), which play a key role in plant growth and development [29]. In plants, ROS act as signaling molecules of ABA and participate in calcium channels and stomatal movement [30]. H_2_O_2_ accumulates during endodormancy and decreases during dormancy release in endodormant floral buds of many plants [31]. This change in H_2_O_2_ content during periods of endodormancy suggests that H_2_O_2_ acts as an important signaling molecule [32]. A transient increase in H_2_O_2_ levels was detected following the application of HC [33], and considered the main reason for accelerated endodormancy release. Interestingly, the relationship between PP2Cs and H_2_O_2_ has been shown during stomatal movement [34]. As a major producer of ROS under stress in plants, plasma membrane-bound NADPH oxidase (RBOH) is suppressed by PP2Cs through SNF1-related protein kinase (SnRK2) [35]. Although it is known that the ABA signaling pathway plays an important role in bud dormancy, specific regulatory networks, such as the PP2C-H_2_O_2_ network, have not been well studied.

Non-coding RNAs (ncRNAs) have recently attracted much attention because of their ability to fine-tune gene expression at the transcriptional or epigenetic level [36]. As one of the main types of ncRNAs, microRNAs (miRNAs) have been widely identified during bud dormancy release by high-throughput sequencing in tree peony (*Paeonia suffruticosa*) [37], *Lilium pumilum* [38] and Japanese pear (*P. pyrifolia* “Kosui”) [39]. In kiwifruit (*Actinidia deliciosa*), miR172 interacts with *APETALA2* (*AP2*) and is involved in dormancy release [40]. In pear, miR6390 [41] and miR156 [42] were predicted to participate in endodormancy release. As another major type of ncRNAs, long non-coding RNAs (lncRNAs, over 200 nucleotides in length) have been suggested to play important roles in developmental stages and stress responses in plants [43]. Interactions between lncRNAs and miRNAs are also widespread. LncRNAs can function as precursors or targets of miRNAs to regulate plant metabolic processes [44]. Although little is known regarding the characteristic features of dormancy-related lncRNAs, the publication of lncRNA data affecting floral bud dormancy in pear plants provides an opportunity for further study of the regulatory mechanism of lncRNAs during endodormancy release [45]. LncRNAs are considered to interact with miRNAs and affect the expression of glutathione peroxidase genes, causing them to participate in the ROS scavenging system during pear bud endodormancy [45]. In plants, ROS are crucial signaling molecules in the ABA-PP2C signaling pathway. Therefore, whether lncRNAs are involved in the regulation of PP2C-H_2_O_2_ network during endodormancy is worth exploring. 

In this study, HC-induced PP2C genes during endodormancy in pear floral buds were identified by RNA-Seq analysis. Through software analysis, the lncRNA *PpL-T31511* was predicted to potentially function as the precursor of *Pp-miRn182*, which targets HC-induced *PpPP2C1*. We investigated this possible lncRNA-miRNA-mRNA regulation mode via fluorescence assays and expression tests. In addition, the promoting effect of ABA treatment on H_2_O_2_ content was found, leading to our investigation of the effect of *PpPP2C1* and *PpL-T31511* on H_2_O_2_ content via transient transfection assay. The results indicated a possible regulatory mechanism of lncRNA *PpL-T31511* in the PP2C-H_2_O_2_ network in pear floral buds during endodormancy, for which we provide a model. 

## 2. Results

### 2.1. HC Treatment Induces Endodormancy Release

**Figure 1:**
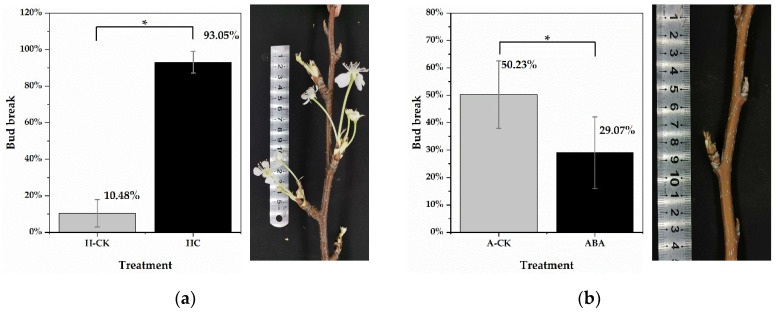
Bud break rate of *P. pyrifolia* “*Huanghua*” after 21 days of treatment. (**a**) The bud break rate of endodormant pear floral buds at 21 days after HC and water (H-CK) treatments. The photo on the right shows the floral bud breakage under HC treatment. (**b**) The bud break rate of ecodormant pear floral buds at 21 days after ABA and water (A-CK) treatments. The photo on the right shows growth arrest of floral buds under ABA treatment. Forty-five shoots from each treatment were selected for bud break tests. Data are presented as the mean ± standard error of three biological replicates; and * indicate significant differences (*p* < 0.05, Tukey’s test) between samples.

In this study, the dormancy status of lateral floral buds was measured on excised one-year-old shoots of “*Huanghua*” pear (*P. pyrifolia*) cultivated in cold storage for 0 days, 15 days, 30 days and 45 days. Lateral floral buds of shoots, cultivated in cold storage for 15 days, had transitioned to endodormancy (10.48% bud breakage was observed under forcing conditions). After adequate chilling accumulation, more than 50% of floral buds had broken on shoots cultivated in cold storage for 45 days (Figure S1). Thus, lateral floral buds of shoots cultivated in cold storage for 45 days were determined to be ecodormant. To identify differentially expressed genes (DEGs), HC treatment was conducted on endodormant floral buds (cultivated in cold storage for 15 days). Twenty-one days post HC treatment, the bud break rate was 93.05% (significantly higher than that in the H-CK group), suggesting that floral buds were induced to endodormancy release (Figure 1a). The bud break rates of ecodormant floral buds (cultivated in cold storage for 45 days) were 29.07% and 50.23% in the ABA treatment group and water treatment group (A-CK), respectively. The results showed that HC treatment effectively stimulated endodormancy release and floral bud breakage in pear plants (Figure 1b). It was noted in the experiment that some pear floral buds began to expand, and even proceeded to burst, under HC treatment after 9 days. Therefore, samples from untreated buds, and those under HC treatment for 0.5 days and 3 days, were selected for subsequent RNA sequencing. Samples treated with water for 0.5 days and 3 days were utilized as controls.

### 2.2. Characterization of the HC-Treated Pear Floral Bud Transcriptome

To search for key genes involved in pear floral buds in response to HC, we performed RNA-Seq analysis. A total of 15 samples, including three biological replicates at each of the three time points (before treatment and 0.5 days and 3 days after treatment) from the HC and H-CK treatment groups, were analyzed. A total of 646,715,158 clean reads were retained after stringent quality assessment and data filtering. By filtering low-quality sequences and those with residual rRNA, approximately 77.04 to 78.66% high-quality clean reads were successfully mapped to the pear genome (Table S1). 

FPKM (fragments per kilobase exon per million mapped fragments) was used to measure gene expression levels in pear floral buds. To confirm the reliability of those expression levels, ten differentially expressed genes were randomly selected for validation and the results were highly consistent with the RNA-Seq data (Figure S2). To explore the HC-responsive genes in pear floral buds, DEGs were screened with absolute fold change value of |log2FC| ≥ 0.5 and false discovery rate (FDR) of ≤0.05. The DEGs that were only induced by HC at 0.5 days (not induced by water treatment) were defined as early HC-induced DEGs, and those that were only induced by HC at 3 days were defined as late HC-induced DEGs. In total, 2336 early and 3527 late HC-induced DEGs were identified (Table 1, Figure S3). It was noted that the number of late HC-induced DEGs was greater than that of early HC-induced DEGs (details for the DEGs can be found in Table S2). 

**Figure 2:**
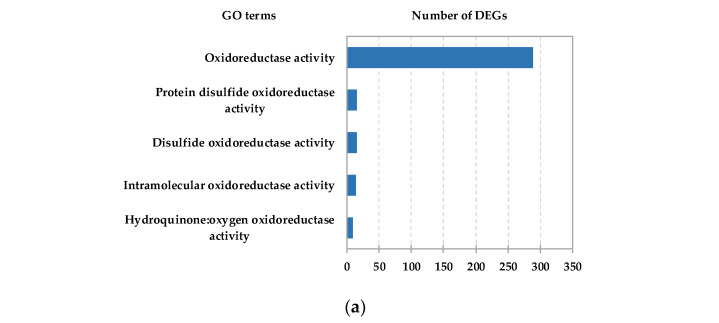

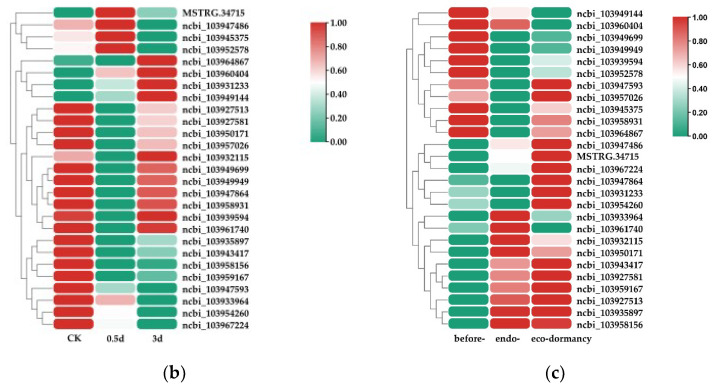
Analysis of DEGs from pear floral buds induced by HC treatment. (**a**) GO analysis of HC responsive DEGs. The most relevant oxidoreductase activity for DEGs is shown; the x-axis represents the number of DEGs. (**b**) The expression heatmap of HC-induced PP2C genes with HC treatment in pear floral buds. (**c**) The expression heatmap of HC-induced PP2C genes during the dormancy process; pear floral buds collected on 27 November 2019 were considered to be pre-dormant, those treated with 4 °C (low temperature) for 15 and 30 days were considered to be endodormant, and those treated with 4 °C (low temperature) for 45 days were considered to be ecodormant.

To further identify the function of HC-induced DEGs, Gene Ontology (GO) term and Kyoto Encyclopedia of Genes and Genomes (KEGG) pathway enrichment analyses of the identified DEGs were carried out. The results showed that large numbers of DEGs were enriched in GO nodes associated with oxidoreductase activity (Figure 2a and Figure S4). Based on KEGG analysis, the “Flavonoid biosynthesis” pathway was identified as one of the top ten enriched pathways in the “Metabolism” category (Figure S4, Table S3). In addition, ROS scavenging system-related DEGs such as the glutathione S-transferase genes (GSTs) and ascorbate peroxidase genes (APXs) were found in the glutathione metabolism pathway (top 20 pathways), suggesting that ROS might be involved in the dormancy release process in pear floral buds. 

Another notable observation was that PP2C genes were identified in the highly enriched “Plant hormone signal transduction” pathway (in the “Environmental Information Processing” category), and the highly enriched “cation binding” GO term. Based on this observation, we screened PP2C genes from the HC-induced DEG set, and a total of 27 PP2C genes were identified. According to the expression trend from the RNA-Seq data, most HC-induced PP2C genes were upregulated by HC treatment (Figure 2b). We also checked the expression of those genes during the dormancy process. The expression heatmap showed that nearly half of the HC-induced PP2C genes were downregulated in endodormancy status and upregulated in endodormancy release status (Figure 2c). Next, we focused on these PP2C genes and studied their possible regulatory relationships with miRNAs or lncRNAs. 

### 2.3. LncRNA PpL-T31511-Derived Pp-miRn182 Targets PpPP2C1 Directly

**Figure 3:**
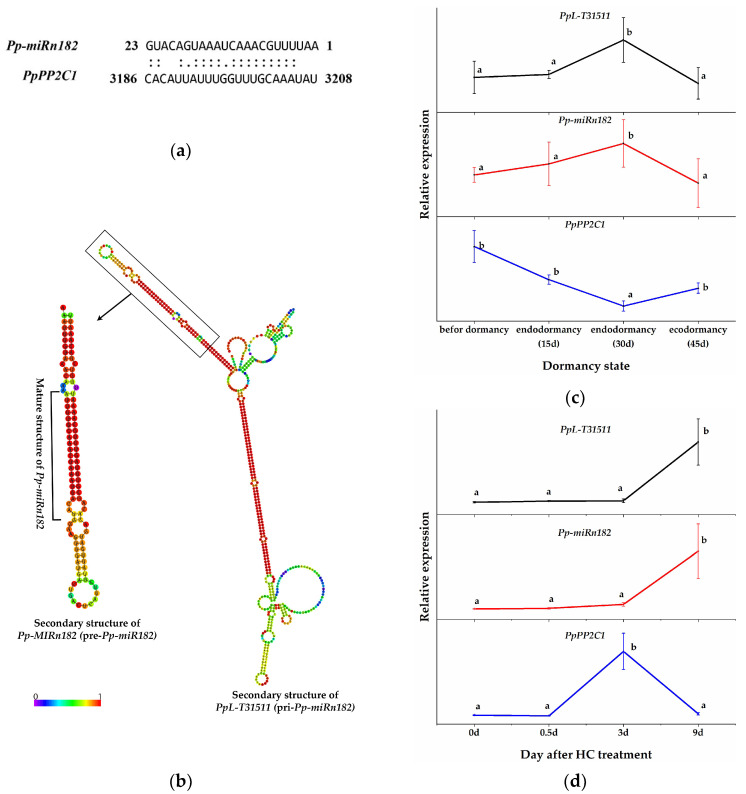
Prediction of the interaction between *Pp-miRn182* and *PpPP2C1*. (**a**) Cleavage site mapping of the *Pp-miRn182* target gene *PpPP2C1*. (**b**) The predicted secondary structure of the *PpL-T31511* (pri-*Pp-miRn182)* and pre-*Pp-miRn182* (*Pp-MIRn182)* sequences. (**c**) The relative expression of *PpL-T31511*, *Pp-miRn182* and *PpPP2C1* during the dormancy process in pear floral buds. (**d**) The relative expression of *PpL-T31511*, *Pp-miRn182* and *PpPP2C1* in pear floral buds treated with HC. Data are presented as the mean ± standard error of three biological replicates; different letters indicate significant differences (*p* < 0.05, Tukey’s test) between samples.

To identify the regulatory relationships between HC-induced PP2C genes and miRNAs in pear plants, the sequences of HC-induced PP2C genes were submitted to the psRNATarget program (Expect ≤ 5) (http://plantgrn.noble.org/psRNATarget/ accessed on 26 October 2021) [46] to predict the target genes of miRNAs (the pear miRNA data were obtained from our previous study [42]). *PpPP2C1* (XM_009364194.2, a transcript of ncbi_103952578) was predicted as the potential target gene of *Pp-miRn182* (a novel pear miRNA, AAUUUUGCAAACUAAAUGACAUG) (Figure 3a). More interestingly, the lncRNA *PpL-T31511* (identified in our previous study [45], NCBI: MW856021) was predicted to contain the functional unit pri-*Pp-miRn182* (Figure 3b). In other words, *PpL-T31511* (pri-*Pp-miRn182*) could be digested properly to form the precursor to *Pp-miRn182* (pre-*Pp-miRn182*, or *Pp-MIRn182*), and eventually to form the mature *Pp-miRn182*. Therefore, we decided to focus our investigation on the interaction between *PpPP2C1*, *Pp-miRn182* and *PpL-T31511*. 

**Figure 4:**
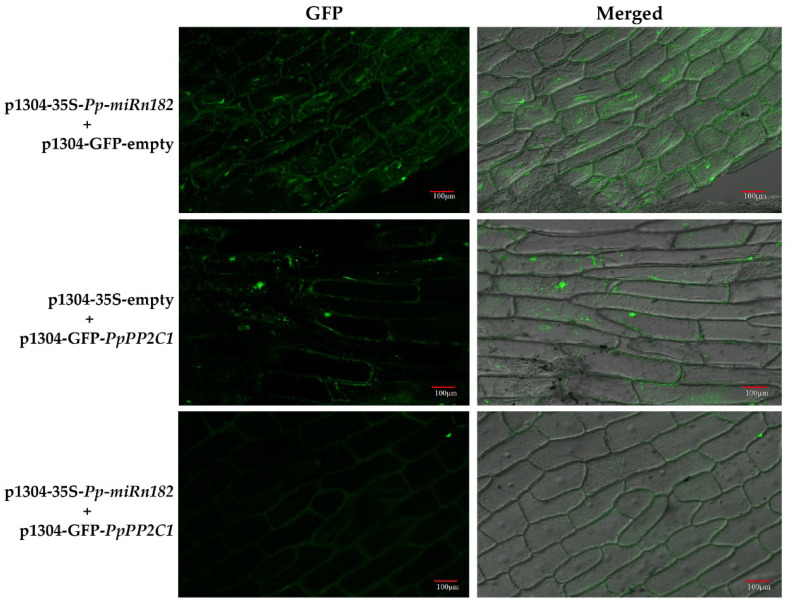
Confirmation of the interactions between *PpPP2C1*, *PpL-T31511* and *Pp-miRn182* from transient expression analysis in onion epidermal cells. In onion epidermal cells co-infected with p1304-35S-*PpL-T31511* and p1304-GFP-empty vectors, it was found that green fluorescence signals appeared in the cell nucleus and cytoplasm, indicating that GFP protein could be expressed in the case of overexpression of *PpL-T31511*. In onion epidermal cells co-infected with p1304-35S-empty and p1304-GFP-*PpPP2C1* vectors, green fluorescence signals were detected in the cell nucleus, indicating that the GFP-*PpPP2C1* fusion protein could be successfully expressed. Few green fluorescence signals were detected in onion epidermal cells co-infected with p1304-35S-*PpL-T31511* and p1304-GFP-*PpPP2C1* vectors, indicating a significant inhibitory effect of *PpL-T31511-*drived *Pp-miRn182* on GFP-*PpPP2C1* fusion protein. Scale bars, 100 µm.

We constructed p1304-35S-*PpL-T31511* and p1304-GFP-*PpPP2C1* vectors. The expression of *Pp-miRn182* was successfully detected in *Nicotiana benthamiana* leaves overexpressing *PpL-T31511*, but not in wild *Nicotiana benthamiana* leaves. We co-infected these two plasmids into onion epidermal cells using the transient transfection assay and detected the fluorescence intensity of the GFP-*PpPP2C1* fusion protein. Confocal microscopic examination revealed lower green fluorescence signals of the GFP-*PpPP2C1* fusion protein in onion epidermis cells with p1304-35S-*PpL-T31511* overexpression than in onion epidermis cells with p1304-35S-empty (control vectors) (Figure 4). This result indicated that the lncRNA *PpL-T31511*-drived *Pp-miRn182* inhibits the expression of the GFP-*PpPP2C1* fusion protein. Next, the expression patterns of *PpPP2C1*, *Pp-miRn182* and *PpL-T31511* in pear floral buds were analyzed. The expression of *PpPP2C1* appeared to increase during endodormancy release, and it could also be induced by HC treatment for 3 days (Figure 3c,d). The expression trends of *Pp-miRn182* and *PpL-T31511* were completely opposite from that of *PpPP2C1*, which was in line with the predicted relationship among them. These results suggested that *PpL-T31511*-derived *Pp-miRn182* targets *PpPP2C1* directly. 

### 2.4. Pp-miRn182-Mediated PpPP2C1 Is Involved in the ABA-PP2C-H_2_O_2_ Pathway

**Figure 5:**
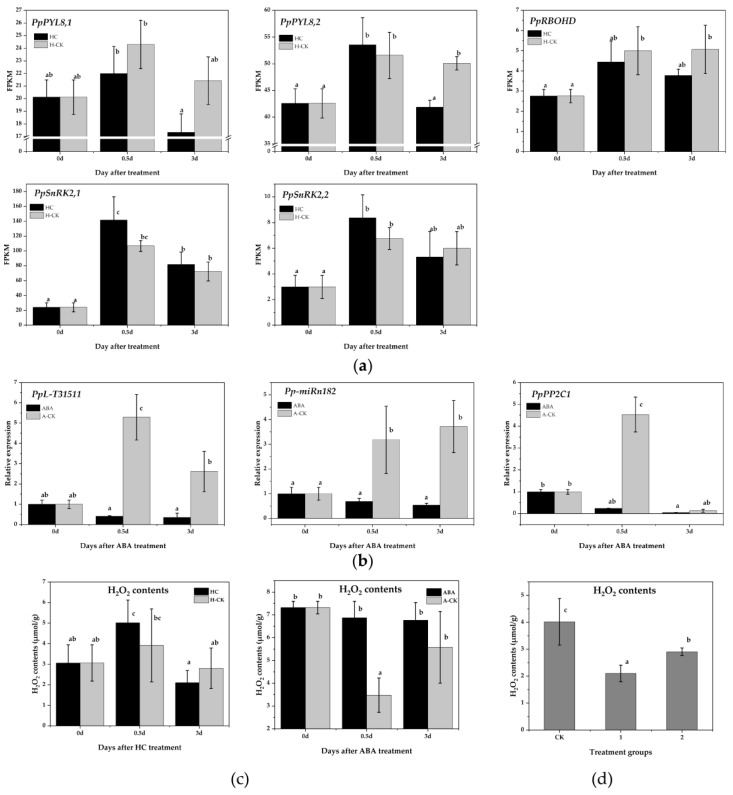
*Pp-miRn182*-mediated *PpPP2C1* is involved in the ABA-H_2_O_2_ pathway. (**a**) The expression of genes related to the ABA signal transduction pathway in pear floral buds with HC treatment from RNA-Seq data. (**b**) qRT-PCR analysis of *PpPP2C1*, *Pp-miRn182* and *PpL-T31511* in pear floral buds under ABA treatment. (**c**) Changes in H_2_O_2_ content after HC and ABA treatments in pear floral buds. (**d**) H_2_O_2_ content in transgenic *Nicotiana benthamiana* leaves overexpressing *PpPP2C1* and/or *PpL-T31511*. CK group represents leaves transiently co-infected with p1304-35S-empty and p1304-MCS35S-empty constructs; group 1 represents leaves transiently co-infected with p1304-35S-empty and p1304-MCS35S-*PpPP2C1* constructs; group 2 represents leaves transiently co-infected with p1304-35S-*PpL-T31511* and p1304-MCS35S-*PpPP2C1* constructs. Data are presented as the mean ± standard error of three biological replicates; different letters indicate significant differences (*p* < 0.05, Tukey’s test) between samples.

PP2Cs are key negative regulatory factors in the ABA signaling network, and form exclusive interactions with PYL (ABA receptors) and SNF1-related protein kinase (SnRK2) [47]. SnRK2 promotes ROS generation by interacting directly with NADPH oxidase (RBOHD) [34]. The ROS scavenging system-related DEGs identified in this study also suggested the possible role of ROS in the endodormancy release response to HC treatment. Therefore, we focused on the ABA-ROS signaling pathway. We first checked the expression of ABA-ROS signaling-related genes in pear plants from the RNA-Seq data. *PpPYL*, *PpSnRK2* and *PpRBOHD* were all downregulated by HC treatment after 3 days, which was opposite that of *PpPP2C1* under HC treatment (Figure 5a). The results indicated a negative interaction between *PpPP2C1* and the ABA signal transduction pathway-related genes.

We subsequently determined the expression of *PpPP2C1*, *Pp-miRn182* and *PpL-T31511* in pear floral buds under ABA treatment. All three were suppressed significantly by ABA, which revealed a more complex regulation of *PpPP2C1* (Figure 5b). Additionally, H_2_O_2_ content was measured in floral buds treated with HC and ABA. The level of H_2_O_2_ increased significantly after 0.5 days of HC treatment and decreased rapidly thereafter. However, the level of H_2_O_2_ in the H-CK group remained stable (Figure 5c). Under ABA treatment, H_2_O_2_ content was maintained at a high level, while it was significantly reduced in the A-CK group at 0.5 days (Figure 5c). These results further validated that ABA inhibits the expression of *PpPP2C1* and enhances the accumulation of H_2_O_2_. 

To check the regulation of *Pp-miRn182* in the PP2C-H_2_O_2_ pathway, we constructed p1304-MCS35S-*PpPP2C1* and p1304-35S-*PpL-T31511* vectors and co-infected them into Nicotiana benthamiana leaves using the transient transfection assay. Then, the H_2_O_2_ content was detected. Compared with CK group leaves (co-infected with p1304-35S-empty and p1304-MCS35S-empty), the H_2_O_2_ contents in group 1 leaves (co-infected with p1304-35S-empty and p1304-MCS35S-*PpPP2C1*) and group 2 leaves (co-infected with p1304-35S-*PpL-T31511* and p1304-MCS35S-*PpPP2C1*) were significantly decreased. In addition, the H_2_O_2_ content in group 2 leaves was higher than that in group 1 leaves (Figure 5d). The results indicated that *PpPP2C1* has a significant inhibitory effect on H_2_O_2_, and *PpL-T31511*-drived *Pp-miRn182* could promote the accumulation of H_2_O_2_ by interacting with *PpPP2C1*.

## 3. Discussion

Bud endodormancy plays a decisive role in the phenology and yield of most plants, especially temperate fruit trees. Insufficient endodormancy in warm winters will result in poor bud quality and lower production in the next growing season [48]. HC is widely used in perennial deciduous fruit trees in warm winter areas as a partial replacement for chilling to effectively release bud endodormancy and promote bud bursting [13]. In addition, HC treatment combined with transcriptome or metabonomics techniques are excellent ways to provide new insights into the mechanism of bud endodormancy release [49,50]. As a result of RNA-Seq analysis, HC was found to induce transient oxidative stress-related genes in peach plants [10]. The increase in TCA metabolites was suggested to be crucial in HC-promoted bud breaking in blueberry plants [9]. The cytokinin pathway was identified as the most upregulated pathway in response to HC during bud dormancy in sweet cherry plants [12]. In this study, HC treatment on endodormant pear floral buds resulted in faster endodormancy release and a higher bud break rate (Figure 1a), as expected, which is similar to the results found in “Kosui” pear plants [11]. During RNA-Seq analysis, 3136 upregulated HC-induced DEGs and 2727 downregulated DEGs were identified. The number of upregulated DEGs is comparable to that of other plants [31]. It can be speculated that more genes participate in bud endodormancy release and bud bursting. Further expression and functional analyses of these DEGs will deepen our understanding of the regulatory network of bud endodormancy. 

Oxidative stress is considered an imperative component of the endodormancy release process [8]. We found that early HC-induced DEGs were more enriched in “oxidoreductase activity”, “oxidation-reduction process” and “oxygen binding” GO terms. In plants, acetaldehyde dehydrogenase (ALDH) genes, the important genes related to oxidoreductase activity, converts acetaldehyde to acetic acid with the byproduct nicotinamide adenine dinucleotide (NADH) [51]. ALDH genes were abundant in the HC-induced DEGs in this study. ALDH is involved in pyruvate metabolism and converts pyruvate during the TCA cycle [52]. The pathways involved in carbohydrate and ATP metabolism in the TCA cycle were stimulated during the endodormancy release phase or by HC treatment to produce enough energy for bud bursting [10,53,54]. It can be speculated that ALDH may accelerate the TCA cycle and endodormancy release by promoting the expression of genes related to these pathways. The TCA cycle-related genes, such as glutamate decarboxylase, aspartate aminotransferase, and acetyl-CoA carboxylase (Table S2), identified in this study may support this. However, what signals or genes control energy metabolism during bud endodormancy? We think that determining this will help us better understand endodormancy. As a proven endogenous hormone that promotes dormancy establishment and maintenance, ABA inhibits TCA cycle isozymes in peach floral buds and affects respiration processes [27]. Reduced TCA cycle enzyme activity and reduced mitochondrial respiration were also found in other plants during endodormancy [55,56]. Notably, ROS production was induced under hypoxic conditions [29]. During dormancy, the increase in the ROS content in buds was attributed to hypoxia and the inhibition of mitochondrial respiration [57]. In our previous study, the decrease in respiratory intensity during endodormancy was examined. We detected a significant decrease in H_2_O_2_ levels after 3 days of HC treatment in this study. However, the balance between TCA cycle activity and ROS production during endodormancy remains unknown. We speculate that the activity of ALDH under HC treatment promotes the TCA cycle, which in turn enhances mitochondrial respiratory intensity, reduces the accumulation of ROS, and ultimately promotes endodormancy release. Therefore, further exploration of the interaction between the TCA cycle and ROS will provide a clearer understanding of the function of oxidative metabolism during endodormancy in plants.

H_2_O_2_ is regarded as a signaling molecule that regulates the dormancy process [58]. The H_2_O_2_ level in dormant buds gradually increased with dormancy onset and decreased rapidly with dormancy release in perennial deciduous trees [31]. Spraying H_2_O_2_ on dormant buds promoted dormancy release in pear [59] and grape [60] plants. Interestingly, a high level of H_2_O_2_ was detected in endodormant and ecodormant buds (A-CK for 0 days, Figure 5c) in this study. The transient increase in H_2_O_2_ levels was attributed to the inhibition of catalase (CAT) by HC treatment [61]. CAT is a crucial enzyme in the ROS scavenging system. This transient increase in the H_2_O_2_ level and inhibition of CAT activity under HC treatment for 0.5 days were detected in our study (Figure S5). After 3 days of HC treatment, H_2_O_2_ content decreased as CAT activity increased. Based on this evidence, we speculated that an appropriately high level of H_2_O_2_ inhibited bud sprouting rather than endodormancy release. The transient increase in H_2_O_2_ levels caused some defense responses in endodormant buds, which accelerated the scavenging of H_2_O_2_ and led to dormancy release and bud sprouting. More evidence is needed to support this hypothesis. 

**Figure 6:**
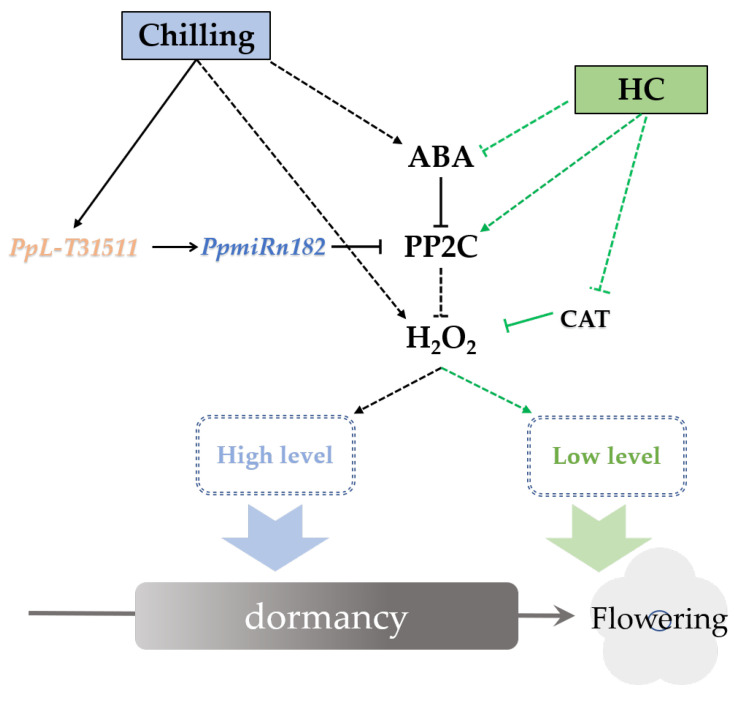
Proposed model of *PpL-T31511*-derived *Pp-miRn182* involvement in the PP2C-H_2_O_2_ pathway in pear endodormant floral buds under HC treatment. Arrows indicate a promoting interaction; T-ends indicate an inhibiting interaction. Dashed lines denote an indirect interaction.

NADPH oxidase (RBOH) is a major producer of ROS in the context of the defense response [62]. ABA promoted the accumulation of H_2_O_2_ by enhancing RBOH activity in the guard cells of leaves. Interestingly, this relationship between ABA and ROS has been reported not only in guard cells but also in dormant seeds [27]. Our study also showed that ABA enhances the accumulation of H_2_O_2_ in endodormant buds. ABA treatment resulted an increase in ABA content (data unpublished) and H_2_O_2_ content (Figure 5c) in pear buds. HC treatment caused a temporary increase followed by a decrease in ABA and H_2_O_2_ content (Figure 5c). This trend also coincided with the expression trends of *PpNCED* and *PpROBH* (Figure 5c and Figure S6). *DAM* was shown to promote ABA content in dormant buds by upregulated expression of *NCED* [11]. We found that the expression of *PpDAM* was continuously inhibited by HC treatment (Figure S6). This inconsistent expression trend of *PpDAM* and *PpNCED* was also observed in “Kousui” pear plants [11]. It could be speculated that the interaction between *PpDAM* and *PpNCED* was conditional. Further study of the relationship between DAM and ABA is required. PP2C is a key negative regulatory factor in the ABA-H_2_O_2_ pathway. The relationship between PP2C and ROS in dormant buds is still unclear. Our results indicated that *PpPP2C1* could act as an RBOH inhibitor, leading to decreased H_2_O_2_ production, and that lncRNA *PpL-T31511*-derived *Pp-miRn182* participated in the regulation of the PP2C-H_2_O_2_ pathway. In addition, we identified a novel miRNA, *Pp-miRn182*, which was formed from the novel lncRNA *PpL-T31511*, and targets *PpPP2C1*. Phytohormone balance was also involved in the induction of endodormancy. Some reports have suggested that a transient spike in ethylene may be a pre-requisite to induction of endodormancy by inducing *NCED1* [63]. The dehydration-responsive element binding protein (DREB)/C-repeat binding factor (CBF) family was identified as a key regulator involved in endodormancy and breakage [63]. DREBs have been reported to positively and negatively mediate ABA responses [64]. A DREB homologous gene was induced by ethylene treatment in leafy spurge, suggesting that the impact of ethylene on DREB family members could play a role in ABA-mediated signaling leading to endodormancy induction [14]. Overexpression of some members of the DREB1 class of AP2/ERF has been shown to result in dwarfed phenotypes through regulating GA metabolism [65]. In this study, some *DERBs* induced by HC were identified. Further study of the regulatory network of phytohormone balance will deepen our understanding of endodormancy. Based on the above results, we propose a hypothetical network model in endodormant pear floral buds (Figure 6). In winter, the accumulation of both ABA and H_2_O_2_ in floral buds is induced under chilling. ABA also upregulates the level of H_2_O_2_ by inhibiting PP2Cs. *PpL-T31511* is induced under chilling and acts as the precursor of *Pp-miRn182* to reduce the expression of *PpPP2C1*. Then, the H_2_O_2_ content in buds reaches a high level, resulting in the establishment of endodormancy. After sufficient chilling accumulation, ABA and *PpL-T31511* are suppressed, and the expression of *PpPP2C1* is upregulated. HC treatment could also inhibit ABA content and promote the expression of *PpPP2C1*, resulting in a rapid decrease of H_2_O_2_ content. In addition, HC induces a transient increase in H_2_O_2_ levels and causes some defense responses in endodormant buds, which accelerates the scavenging of H_2_O_2_. With the decrease in H_2_O_2_, floral buds release from endodormancy. 

While this proposed network model needs more experimental evidence, it suggests a possible role of lncRNAs in the PP2C-H_2_O_2_ pathway during endodormancy release. In future work, the establishment of a suitable genetic transformation system for pear plants will enable exploration of the function of lncRNAs. In addition, miRNA *Pp-miRn182* was specifically upregulated during endodormancy in pear floral buds, suggesting an important role in the endodormancy process. A total of 160 transcripts were identified as target genes for *Pp-miRn182* in pear plants, including glutamate dehydrogenase, galactosyltransferase and MYB (Table S4). Thus, the study of more *Pp-miRn182* target genes will help to fully reveal the regulatory network of *Pp-miRn182* in the endodormancy process. Furthermore, the specific roles of lncRNA *PpL-T31511*, except for its role as a precursor of *Pp-miRn182*, in endodormant pear buds are still unknown. Further study on *PpL-T31511* would be helpful to explore the regulatory mechanism of lncRNAs during the dormancy process.

## 4. Conclusions

In summary, we performed RNA-Seq analysis on pear endodormant floral buds under HC treatment and found 27 HC-induced PP2C genes. LncRNA *PpL-T31511* was identified as the precursor of *Pp-miRn182*, which targets the HC-induced PP2C gene *PpPP2C1*. Expression analysis demonstrated that *PpPP2C1* was induced by HC treatment. Transient assays showed that overexpression of *PpPP2C1* repressed the production of H_2_O_2_. Moreover, a high level of H_2_O_2_ was shown to be an inhibitor of bud sprouting. Our study suggests that the *PpL-T31511*-*PpPP2C1*-H_2_O_2_ may play an important role in the endodormancy process and bud bursting.

## 5. Materials and Methods

### 5.1. Plant Material and Treatment

Ten-year-old pear trees (“*Huanghua*”, *P. pyrifolia*) were grown in an experimental orchard in Gaozhen Village, Jianning Town, Sanming City, Fujian Province, China (altitude, 318 m; latitude, 26°84′37″ N; and longitude, 116°81′19″ E). The yields of these pear trees were stable, and they were regarded as being in the adult phase. These trees were not pruned or chemically treated for more than a month before sampling. One-year-old shoots (approximately 50–80 cm long and bearing 8–15 lateral floral buds) of pear trees were collected on 27 November 2019 (based on production experience, lateral floral buds were in paradormancy during this period) and placed in water in vials in cold storage (temperature: 4 ± 1 °C relative humidity: 75%). The water in the vials was changed every seven days. After 15 days, 30 days and 45 days, the shoots were removed from cold storage in batches in order to estimate the dormancy status of lateral floral buds and the follow-up treatments. 

Forty-five shoots each cultured in cold storage for 15 days, 30 days, and 45 days were collected, and placed in water in vials in a phytotron (kept under conditions of day/night: 14 h/10 h, temperature: 25 ± 1/20 ± 1 °C relative humidity 75%). The base of the shoot was trimmed, and the water in the vials was changed every 3–5 days. After 21 days, the percentage of bud breakage was determined to evaluate the dormancy status (Figure S7). Green leaf tips enclosing visible flowers were used as a sign of bud breakage [66]. Lateral floral buds of shoots after 21 days of cultivation with bud break rates ≥ 50% were defined as being in the endodormancy release stage [67].

### 5.2. HC and ABA Treatments

Based on our previous study [45] and the results of this study (Figure S1), the lateral floral buds cultivated in water in cold storage for 15 days were determined as being in the endodormancy phase. Accordingly, the shoots cultivated in water in cold storage for 15 days were transferred to the lab. 1% *w*/*v* HC solution (Shengruisibio, Qingdao, China) or water (defined as the H-CK group) was applied to lateral floral buds. After treatments, the shoots were placed in water in a phytotron (kept under a day/night: 14 h/10 h, temperature: 25 ± 1/20 ± 1 °C, relative humidity 75%). Three replicates were performed for each treatment. Each biological replicate contained about 300 shoots. The base of the shoot was trimmed, and the water in the flask was changed every 3–5 days. The percentage of bud breakage was determined at 21 days after treatment. Forty-five shoots from each treatment were selected for the bud break test. Lateral floral buds were collected from HC-treated and water-treated shoots at 0.5 days, 3 days and 9 days after treatment. Lateral floral buds before treatment were also sampled as a control. At every sampling point, more than 50 shoots (approximately 500 floral buds) were harvested from each replicate group for different measurements. After peeling off the external scales of the buds, floral bud primordium tissues were frozen immediately in liquid nitrogen and stored at −80 °C until analyzed.

To further study the regulatory network between candidate genes, the shoots cultivated in water in cold storage for 45 days (were determined as being in the ecodormancy phase, see Figure S1) were transferred to the lab and treated with 150 mg/L ABA (Solarbio, Beijing, China) or water (defined as the A-CK group). The cultivation conditions and sampling points were the same as those in the HC treatment above.

### 5.3. RNA Extraction, Library Construction and Sequencing

For RNA-Seq analysis, three biological replicates from each of the following sample points were selected: the floral buds before HC treatment (named CK), 0.5 days and 3 days after HC treatment (named as T1 and T2), and 0.5 days and 3 days after water treatment (named TCK1 and TCK2). Total RNA from each sample was extracted with a TRIzol reagent kit (Invitrogen, Carlsbad, CA, USA) according to the manufacturer’s instructions. The stand-specific RNA library was constructed by using the NEB#E7530 Ultra RNA library prep kit from Illumina (NEB, Ipswich, MA, USA). Then, the cDNA fragments were purified with a QiaQuick PCR extraction kit (Qiagen, Venlo, Netherlands) and ligated into Illumina sequencing adapters. After agarose gel electrophoresis, suitable fragments were selected as templates for PCR amplification, and sequencing was carried out by Gene Denovo Biotechnology Co. (Guangzhou, China; http://www.genedenovo.com/, accessed on 18 March 2020) on an Illumina HiSeq2500 system (paired-end reads were generated). 

### 5.4. Bioinformatics Analyses

Raw reads obtained from the Illumina HiSeq2500 system were further filtered to exclude low-quality reads and reads containing adaptor sequences. The resulting high-quality clean reads were aligned to pear ribosome data (ftp://ftp.ncbi.nlm.nih.gov/genbank/, accessed on 18 March 2020) using Bowtie 2 (2.2.8) (local alignment; E ≤ 10–5), and those that mapped to rRNA were removed. The remaining reads were further aligned to the pear reference genome (from NCBI: https://www.ncbi.nlm.nih.gov/genome/12793, accessed on 18 March 2020) using HISAT2.2.4 with “-rna-strandness RF” and other parameters set to default. The mapped reads of each sample were assembled by using StringTie (version 1.3.1) [68,69] in a reference-based approach. FPKM values were utilized to normalize the expression of each gene. Differentially expressed genes (DEGs) were identified between pairs of samples (false discovery rate (FDR) ≤ 0.05 and |log2FC| ≥ 0.5) with DESeq2 software. The DEGs that were only induced by HC at 0.5 days or 3 days were defined as HC-induced DEGs. Sequencing data were uploaded into the NCBI Sequence Read Archive (SRA accession number: PRJNA715605). 

### 5.5. Functional Enrichment Analyses

The HC-induced DEGs in this study were submitted to OmicShare online tools (www.omicshare.com/tools, accessed on 4 April 2020) for GO term and KEGG enrichment analysis. Pear background files of GO terms and KEGG pathways were obtained from the GDR database (The Genome Database for Rosaceae, https://www.rosaceae.org, accessed on 6 April 2020) [70]. The calculated *p*-value was subjected to FDR correction, taking FDR ≤ 0.05 as a threshold. GO terms and KEGG pathways meeting this condition were defined as significantly enriched GO terms and KEGG pathways. 

### 5.6. Prediction of the Regulatory Relationship between HC-Induced PP2Cs, miRNAs and lncRNAs

The PP2Cs screened from the HC-induced DEG sets were defined as HC-induced PP2Cs. “*Huanghua*” pear miRNA and lncRNA data were obtained from our previous study [42,45]. The HC-induced PP2Cs and pear miRNA sequences were submitted to the psRNATarget program (Expect ≤ 5) (http://plantgrn.noble.org/psRNATarget/, accessed on 23 April 2020) [46] to predict the candidate miRNAs that target HC-induced PP2Cs. The lncRNA sequences were aligned to miRBase (version 21) [71] using BLAST to identify miRNA precursors. Sequences with greater than 90% alignment were considered precursors of miRNAs. 

### 5.7. Vector Construction

Total RNA were extracted (TRIzol reagent kit) from the same samples for RNA-Seq. The sequences of *PpPP2C1* and *PpL-T31511* were obtained for “*Huanghua*” pear by using a *FastPfu* SurperMix clone kit (TransGen Biotech, Beijing, China). Different vectors were used for specific purposes. To validate the interaction of miRNA and genes, two vectors pNC-Cam1304-35S (used to overexpress target genes, and does not contain any fluorescently labeled protein) and pNC-Cam1304-SubN (used to construct GFP-target gene fusion protein, target genes can be inserted after GFP protein) were selected. To construct the *PpL-T31511* overexpression vector, part of the *PpL-T31511* sequence (including the *Pp-miRn182* precursor) was inserted into the NC frame of pNC-Cam1304-35S by using a Nimble Cloning kit (Genepioneer, Nanjing, China) [72]. To construct the GFP-*PpPP2C1* fusion protein, the sequence of *PpPP2C1* was inserted into the NC frame of pNC-Cam1304-SubN by the same method. The recombinant vectors were designated p1304-35S-*Pp-miRn182* and p1304-GFP-*PpPP2C1*. The vector pNC-Cam1304-MCS35S (used to overexpress target genes, contain GFP and GUS labeled protein) was utilized in transient transgenic plant construction for *PpPP2C1* overexpressing (Figure S8). The recombinant vector was designated p1304-MCS35S-*PpPP2C1*. The primers used for gene amplification and vector construction were designed with Primer5 software and were synthesized commercially (BioSune, Shanghai, China) (details of the primers can be found in Table S5). All constructs were individually transformed into *Agrobacterium tumefaciens* GV3101 using the heat shock method.

### 5.8. Transient Assays

Transient assays were performed with *Nicotiana benthamiana* leaves for *Pp-miRn182* validation and H_2_O_2_ detection. To confirm precursors of *Pp-miRn182*, the p1304-35S-*Pp-miRn182* construct and p1304-35S-empty (as control) were individually infected into *Nicotiana benthamiana* leaves via *Agrobacterium*-mediated transformation. At 3 days after infection, the expression of *Pp-miRn182* was detected via PCR. To study the regulation between PP2C and H_2_O_2_, p1304-35S-*Pp-miRn182* + p1304-MCS35S-*PpPP2C1* constructs were co-infected into *Nicotiana benthamiana* leaves, and H_2_O_2_ content was detected after 3 days. The p1304-35S-empty + p1304-MCS35S-empty constructs and p1304-35S-empty + p1304-MCS35S-*PpPP2C1* constructs were also infected as controls. H_2_O_2_ content was measured three times for every experimental condition.

For the fluorescence assay, onion epidermal cells were infected with p1304-35S-*Pp-miRn182* and/or p1304-GFP-*PpPP2C1*. Three days after infection, GFP-*PpPP2C1* expression was analyzed using an FV1200 laser scanning microscope (OLYMPUS, Tokyo, Japan). GFP signals were collected using an emission filter BP505-530 nm with an excitation at 488 nm. 

### 5.9. Measurement of H_2_O_2_ Content

H_2_O_2_ content was examined using a detection kit (H_2_O_2_-1-Y, Comin Biotechnology Co., Suzhou, China) based on appropriate improvements. Approximately 0.05 g of fresh plant tissue was quickly homogenized in acetone solution and centrifuged at 8000× *g* for 10 min at 4 °C Then, 100 μL of 5% titanium sulfate and 100 μL of concentrated ammonia water were added to the supernatant (with acetone solution as a control) and centrifuged at 4000× *g* for 10 min at 25 °C. The precipitate was extracted, and 1 mL of 2 mol/L sulfuric acid was added to fully dissolve the precipitate. The solution was held at 25 °C for 5 min, after which absorption was measured at 415 nm. In addition, CAT activity was examined using a detection kit (CAT-1-W, Comin Biotechnology Co., Suzhou, China). 

### 5.10. Quantitative Real-Time PCR for Validation

To confirm the RNA-Seq results, ten differentially expressed genes were randomly selected for validation. The expression levels of *PpPP2C1*, *PpL-T31511* and *Pp-miRn182* in pear floral buds under HC and ABA treatment were also analyzed. Total RNA and miRNA from the samples of HC and ABA treatments (including three replicated samples for each treatment) were extracted, respectively. cDNAs synthesis of total RNA and miRNA were performed according to TransScript miRNA First-Strand cDNA Synthesis SuperMix (TransGen, Beijing, China) Instruction Manual, respectively. Twenty-fold dilutions of cDNAs were used as templates for quantitative real-time PCR (qRT-PCR). qRT-PCR was performed using a Top Green qPCR SuperMix kit (TransGen, Beijing, China) on a Roche LightCycler 96 system (Roche, Basel, Switzerland), according to the manufacturer’s instructions. Quantification results were calculated using the relative quantification method (2^−ΔΔCt^). The transcript levels of *PpPP2C1*, *PpL-T31511* and ABA-ROS signaling-related genes were normalized to that of *PpActin*, which has been widely used as an internal reference gene for pear plants [66]. The transcript levels of *Pp-miRn182* was normalized to that of *5S* rRNA, which has proved to be highly stable in our previous studies on pear miRNAs [73]. All primers were designed with Primer5 software and were synthesized commercially (BioSune, Shanghai, China) (details of the primers can be found in Table S5). 

### 5.11. Statistical Analyses

Each experiment was repeated at least three times. The significance of between-group differences was analyzed using Tukey’s test. All statistical analyses were performed using IBM SPSS software. *p* < 0.05 was considered to be statistically significant.

## Figures and Tables

**Table 1:** Number of HC responsive DEGs.

DEG Set	DEG Number	Upregulated	Downregulated
Early HC-induced DEGs	2336	1119	1217
Late HC-induced DEGs	3527	2017	1510

## Data Availability

RNA sequencing data are available from the NCBI Sequence Read Archive (SRA) database (SRA accession number: PRJNA715605; https://trace.ncbi.nlm.nih.gov/Traces/sra/sra.cgi? accessed on 26 October 2021).

## Supplementary Materials

The following are available online at https://www.mdpi.com/article/10.3390/ijms222111842/s1.

## References

[1] Rohde A., Bhalerao R.P. (2007). Plant dormancy in the perennial context. Trends Plant Sci..

[2] Lang G.A., Early J.D., Martin G.C., Darnell R.L. (1987). Endo-, para-, and eco-dormancy: Physiological terminology and classification for dormancy research. HortScience.

[3] Liu Z., Zhu H., Abbott A., Anderson J.V. (2015). Dormancy Behaviors and Underlying Regulatory Mechanisms: From Perspective of Pathways to Epigenetic Regulation. Advances in Plant Dormancy.

[4] Rohde A., Ruttink T., Hostyn V., Sterck L., Van Driessche K., Boerjan W. (2007). Gene expression during the induction, maintenance, and release of dormancy in apical buds of poplar. J. Exp. Bot..

[5] Heide O.M., Prestrud A.K. (2005). Low temperature, but not photoperiod, controls growth cessation and dormancy induction and release in apple and pear. Tree Physiol..

[6] Cooke J.E.K., Eriksson M.E., Junttila O. (2012). The dynamic nature of bud dormancy in trees: Environmental control and molecular mechanisms. Plant Cell Environ..

[7] Li J., Yan X., Yang Q., Ma Y., Yang B., Tian J., Teng Y., Bai S. (2019). PpCBFs selectively regulate *PpDAMs* and contribute to the pear bud endodormancy process. Plant Mol. Biol..

[8] Rehman M., Wang W., Dong Y., Faheem M., Xu Y., Gao Z., Shen Z., Tao J. (2019). Comparative Transcriptomic and Proteomic Analysis to Deeply Investigate the Role of Hydrogen Cyanamide in Grape Bud Dormancy. Int. J. Mol. Sci..

[9] Wang H., Xia X., An L. (2021). Metabolomics Analysis Reveals the Mechanism of Hydrogen Cyanamide in Promoting Flower Bud Break in Blueberry. Agronomy.

[10] Tang L., Chhajed S., Vashisth T., Olmstead M., Olmstead J., Colquhoun T. (2019). Transcriptomic Study of Early Responses to the Bud Dormancy-breaking Agent Hydrogen Cyanamide in ‘TropicBeauty’ Peach. J. Am. Soc. Hortic. Sci..

[11] Tuan P.A., Bai S., Saito T., Ito A., Moriguchi T. (2017). *Dormancy-Associated MADS-Box* (*DAM*) and the Abscisic Acid Pathway Regulate Pear Endodormancy Through a Feedback Mechanism. Plant Cell Physiol..

[12] Ionescu I.A., López-Ortega G., Burow M., Bayo-Canha A., Junge A., Gericke O., Møller B.L., Sánchez-Pérez R. (2017). Transcriptome and Metabolite Changes during Hydrogen Cyanamide-Induced Floral Bud Break in Sweet Cherry. Front. Plant Sci..

[13] Liang D., Huang X., Shen Y., Shen T., Zhang H., Lin L., Deng Q., Lyu X., Xia H. (2019). Hydrogen cyanamide induces grape bud endodormancy release through carbohydrate metabolism and plant hormone signaling. BMC Genom..

[14] Doğramacı M., Foley M.E., Chao W.S., Christoffers M.J., Anderson J.V. (2013). Induction of endodormancy in crown buds of leafy spurge (*Euphorbia esula* L.) implicates a role for ethylene and cross-talk between photoperiod and temperature. Plant Mol. Biol..

[15] Chao W.S., Doğramaci M., Horvath D.P., Anderson J.V., Foley M.E. (2016). Phytohormone balance and stress-related cellular responses are involved in the transition from bud to shoot growth in leafy spurge. BMC Plant Biol..

[16] Parada F., Noriega X., Dantas D., Bressan-Smith R., Pérez F.J. (2016). Differences in respiration between dormant and non-dormant buds suggest the involvement of ABA in the development of endodormancy in grapevines. J. Plant Physiol..

[17] Khalil-Ur-Rehman M., Wang W., Xu Y.-S., Haider M.S., Li C.-X., Tao J.-M. (2017). Comparative Study on Reagents Involved in Grape Bud Break and Their Effects on Different Metabolites and Related Gene Expression during Winter. Front. Plant Sci..

[18] Li J., Xu Y., Niu Q., He L., Teng Y., Bai S. (2018). Abscisic Acid (ABA) Promotes the Induction and Maintenance of Pear (*Pyrus pyrifolia* White Pear Group) Flower Bud Endodormancy. Int. J. Mol. Sci..

[19] Wang D., Gao Z., Du P., Xiao W., Tan Q., Chen X., Li L., Gao D. (2015). Expression of ABA Metabolism-Related Genes Suggests Similarities and Differences Between Seed Dormancy and Bud Dormancy of Peach (*Prunus persica*). Front. Plant Sci..

[20] Prudencio Á.S., Dicenta F., Martínez-Gómez P. (2018). Monitoring Dormancy Transition in Almond [*Prunus Dulcis* (Miller) Webb] during Cold and Warm Mediterranean Seasons through the Analysis of a *DAM* (*Dormancy-Associated MADS-Box*) Gene. Horticulturae.

[21] Balogh E., Halász J., Soltész A., Erös-Honti Z., Gutermuth Á., Szalay L., Höhn M., Vágújfalvi A., Galiba G., Hegedüs A. (2019). Identification, Structural and Functional Characterization of Dormancy Regulator Genes in Apricot (*Prunus armeniaca* L.). Front. Plant Sci..

[22] Umezawa T., Sugiyama N., Mizoguchi M., Hayashi S., Myouga F., Yamaguchi-Shinozaki K., Ishihama Y., Hirayama T., Shinozaki K. (2009). Type 2C protein phosphatases directly regulate abscisic acid-activated protein kinases in *Arabidopsis*. Proc. Natl. Acad. Sci. USA.

[23] Nishimura N., Yoshida T., Murayama M., Asami T., Shinozaki K., Hirayama T. (2004). Isolation and characterization of novel mutants affecting the abscisic acid sensitivity of *Arabidopsis* germination and seedling growth. Plant Cell Physiol..

[24] Nishimura N., Tsuchiya W., Moresco J.J., Hayashi Y., Satoh K., Kaiwa N., Irisa T., Kinoshita T., Schroeder J.I., Yates J.R. (2018). Control of seed dormancy and germination by DOG1-AHG1 PP2C phosphatase complex via binding to heme. Nat. Commun..

[25] Née G., Kramer K., Nakabayashi K., Yuan B., Xiang Y., Miatton E., Finkemeier I. (2017). DELAY OF GERMINATION1 requires PP2C phosphatases of the ABA signalling pathway to control seed dormancy. Nat. Commun..

[26] Yu X., Han J., Li L., Zhang Q., Yang G.X., He G. (2020). Wheat PP2C-a10 regulates seed germination and drought tolerance in transgenic Arabidopsis. Plant Cell Rep..

[27] Beauvieux R., Wenden B., Dirlewanger E. (2018). Bud dormancy in perennial fruit tree species: A pivotal role for oxidative cues. Front. Plant Sci..

[28] Tan Y., Li L., Leng C.-Y., Li D., Chen X., Gao D. (2013). Respiratory Response of Dormant Nectarine Vegetative Buds to High Temperature Stress. J. Integr. Agric..

[29] Considine M.J., Foyer C.H. (2014). Redox regulation of plant development. Antioxid. Redox Signal..

[30] Kwak J.M., Nguyen V., Schroeder J.I. (2006). The Role of Reactive Oxygen Species in Hormonal Responses. Plant Physiol..

[31] Liu F., Liang D., Lv X., Gong M. (2017). The Effect of Promoting Sprouting Process by Cyanamide on the Growth of Grape Leaves. J. Biobased Mater. Bioenergy.

[32] Hussain S., Niu Q., Yang F., Hussain N., Teng Y. (2015). The possible role of chilling in floral and vegetative bud dormancy release in *Pyrus pyrifolia*. Biol. Plant..

[33] Sudawan B., Chang C.-S., Chao H.-F., Ku M.S.B., Yen Y.-F. (2016). Hydrogen cyanamide breaks grapevine bud dormancy in the summer through transient activation of gene expression and accumulation of reactive oxygen and nitrogen species. BMC Plant Biol..

[34] Mittler R., Blumwald E. (2015). The roles of ROS and ABA in systemic acquired acclimation. Plant Cell.

[35] Raghavendra A.S., Gonugunta V.K., Christmann A., Grill E. (2010). ABA perception and signalling. Trends Plant Sci..

[36] Robinson E., Covarrubias S., Carpenter S. (2019). The how and why of lncRNA function: An innate immune perspective. Biochim. Biophys. Acta BBA Gene Regul. Mech..

[37] Zhang Y., Wang Y., Gao X., Liu C., Gai S. (2018). Identification and characterization of microRNAs in tree peony during chilling induced dormancy release by high-throughput sequencing. Sci. Rep..

[38] Zhou Y., Wang W., Yang L., Su X., He M. (2021). Identification and Expression Analysis of microRNAs in Response to Dormancy Release During Cold Storage of *Lilium pumilum* Bulbs. J. Plant Growth Regul..

[39] Bai S., Saito T., Ito A., Tuan P.A., Xu Y., Teng Y., Moriguchi T. (2016). Small RNA and PARE sequencing in flower bud reveal the involvement of sRNAs in endodormancy release of Japanese pear (*Pyrus pyrifolia* ‘Kosui’). BMC Genom..

[40] Varkonyi-Gasic E., Lough R.H., Moss S.M.A., Wu R., Hellens R.P. (2012). Kiwifruit floral gene *APETALA2* is alternatively spliced and accumulates in aberrant indeterminate flowers in the absence of miR172. Plant Mol. Biol..

[41] Niu Q., Li J., Li J., Cai D., Qian M., Jia H., Bai S., Hussain S., Liu G., Teng Y. (2016). Dormancy-associated MADS-box genes and microRNAs jointly control dormancy transition in pear (*Pyrus pyrifolia* white pear group) flower bud. J. Exp. Bot..

[42] Ma X., Li L., Liu J., Yang M., Chen J., Liang Q., Wu S., Li Y. (2018). Identification and Differentially Expressed Analysis of microRNA Associated with Dormancy of Pear Flower Buds. Acta Hortic. Sin..

[43] Budak H., Biyiklioglu-Kaya S., Cagirici H. (2020). Long Non-coding RNA in Plants in the Era of Reference Sequences. Front. Plant Sci..

[44] Chen Y., Li X., Su L., Chen X., Zhang S., Xiaoping X., Zhang Z., Chen Y., XuHan X., Lin Y. (2018). Genome-wide identification and characterization of long non-coding RNAs involved in the early somatic embryogenesis in *Dimocarpus longan* Lour. BMC Genom..

[45] Li L., Liu J., Liang Q., Zhang Y., Kang K., Wang W., Feng Y., Wu S., Yang C., Li Y. (2021). Genome-wide analysis of long noncoding RNAs affecting floral bud dormancy in pears in response to cold stress. Tree Physiol..

[46] Dai X., Zhao P.X. (2011). psRNATarget: A plant small RNA target analysis server. Nucleic Acids Res..

[47] Soon F.-F., Ng L.-M., Zhou X.E., West G.M., Kovach A., Tan M.H.E., Suino-Powell K.M., He Y., Xu Y., Chalmers M.J. (2012). Molecular mimicry regulates ABA signaling by SnRK2 kinases and PP2C phosphatases. Science.

[48] Campoy J., Ruiz D., Egea J. (2011). Dormancy in temperate fruit trees in a global warming context: A review. Sci. Hortic..

[49] Vergara R., Noriega X., Parada F., Dantas D., Pérez F.J. (2016). Relationship between endodormancy, *FLOWERING LOCUS T* and cell cycle genes in *Vitis vinifera*. Planta.

[50] Wu R., Walton E., Richardson A., Wood M., Hellens R., Varkonyi-Gasic E. (2011). Conservation and divergence of four kiwifruit *SVP*-like MADS-box genes suggest distinct roles in kiwifruit bud dormancy and flowering. J. Exp. Bot..

[51] Kang J.H., Lee S.-H., Lee J.-S., Nam B., Seong T.W., Son J., Jang H., Hong K.M., Lee C., Kim S.-Y. (2016). Aldehyde dehydrogenase inhibition combined with phenformin treatment reversed NSCLC through ATP depletion. Oncotarget.

[52] Stiti N., Giarola V., Bartels D. (2021). From algae to vascular plants: The multistep evolutionary trajectory of the ALDH superfamily towards functional promiscuity and the emergence of structural characteristics. Environ. Exp. Bot..

[53] Zhang T., Yuan Y., Zhan Y., Cao X., Liu C., Zhang Y., Gai S. (2020). Metabolomics analysis reveals Embden Meyerhof Parnas pathway activation and flavonoids accumulation during dormancy transition in tree peony. BMC Plant Biol..

[54] Horikoshi H.M., Sekozawa Y., Kobayashi M., Saito K., Kusano M., Sugaya S. (2018). Metabolomics analysis of ‘Housui’ Japanese pear flower buds during endodormancy reveals metabolic suppression by thermal fluctuation. Plant Physiol. Biochem..

[55] Considine M., Díaz-Vivancos P., Kerchev P., Signorelli S., Agudelo-Romero P., Gibbs D., Foyer C. (2016). Learning to Breathe: Developmental Phase Transitions in Oxygen Status. Trends Plant Sci..

[56] Murcia G., Pontin M., Reinoso H., Baraldi R., Bertazza G., Gómez-Talquenca S., Bottini R., Piccoli P.N. (2016). ABA and GA3 increase carbon allocation in different organs of grapevine plants by inducing accumulation of non-structural carbohydrates in leaves, enhancement of phloem area and expression of sugar transporters. Physiol. Plant..

[57] Meitha K., Agudelo-Romero P., Signorelli S., Gibbs D.J., Considine J.A., Foyer C.H., Considine M.J. (2018). Developmental control of hypoxia during bud burst in grapevine. Plant Cell Environ..

[58] Wang Z., Ma R., Zhao M., Wang F., Zhang N., Si H. (2020). NO and ABA Interaction Regulates Tuber Dormancy and Sprouting in Potato. Front. Plant Sci..

[59] Kuroda H., Sugiura T., Sugiura H. (2005). Effect of Hydrogen Peroxide on Breaking Endodormancy in Flower Buds of Japanese Pear (*Pyrus pyrifolia* Nakai). Engei Gakkai Zasshi.

[60] Pérez F., Vergara R., Rubio S. (2008). H_2_O_2_ is involved in the dormancy-breaking effect of hydrogen cyanamide in grapevine buds. Plant Growth Regul..

[61] Tan Y., Ling L.I., Xu C., Chen X., Li D., Gao D. (2012). Possible involvement of H_2_O_2_ induced Ca^2+^ efflux in the dormancy-breaking effect of hydrogen cyanamide and high temperature in nectarine floral buds. J. Food Agric. Environ..

[62] Davies D., Bindschedler L., Strickland T., Bolwell G. (2006). Production of reactive oxygen species in *Arabidopsis thaliana* cell suspension cultures in response to an elicitor from *Fusarium oxysporum*: Implications for basal resistance. J. Exp. Bot..

[63] Doğramacı M., Horvath D.P., Christoffers M.J., Anderson J.V. (2011). Dehydration and vernalization treatments identify overlapping molecular networks impacting endodormancy maintenance in leafy spurge crown buds. Funct. Integr. Genom..

[64] Xu Z.S., Chen M., Li L.C., Ma Y.Z. (2011). Functions and application of the AP2/ERF transcription factor family in crop improvement. J. Integr. Plant Biol..

[65] Doğramaci M., Anderson J.V., Chao W.S., Horvath D.P., Hernandez A.G., Mikel M.A., Foley M.E. (2017). Foliar Glyphosate Treatment Alters Transcript and Hormone Profiles in Crown Buds of Leafy Spurge and Induces Dwarfed and Bushy Phenotypes throughout its Perennial Lifecycle. Plant Genome.

[66] Liu G., Li W., Zheng P., Xu T., Chen L., Liu D., Hussain S., Teng Y. (2012). Transcriptomic analysis of ‘Suli’ pear (*Pyrus pyrifolia* white pear group) buds during the dormancy by RNA-Seq. BMC Genom..

[67] Sugiura T., Kuroda H., Honjo H., Ito D. (2002). Temperature dependence of endodormancy development in flower buds of ‘kousui’ japanese pear and a model for estimating the completion of endodormancy. Acta Hortic..

[68] Pertea M., Kim D., Pertea G.M., Leek J.T., Salzberg S.L. (2016). Transcript-level expression analysis of RNA-seq experiments with HISAT, StringTie and Ballgown. Nat. Protoc..

[69] Pertea M., Pertea G.M., Antonescu C.M., Chang T.C., Mendell J.T., Salzberg S.L. (2015). StringTie enables improved reconstruction of a transcriptome from RNA-seq reads. Nat. Biotechnol..

[70] Jung S., Lee T., Cheng C.H., Buble K., Zheng P., Yu J., Humann J., Ficklin S.P., Gasic K., Scott K. (2019). 15 years of GDR: New data and functionality in the Genome Database for Rosaceae. Nucleic Acids Res..

[71] Griffiths-Jones S., Grocock R.J., Van Dongen S., Bateman A., Enright A.J. (2006). miRBase: microRNA sequences, targets and gene nomenclature. Nucleic Acids Res..

[72] Yan P., Zeng Y., Shen W., Tuo D., Li X., Zhou P. (2020). Nimble Cloning: A Simple, Versatile, and Efficient System for Standardized Molecular Cloning. Front. Bioeng. Biotechnol..

[73] Shi R., Chiang V.L. (2005). Facile means for quantifying microRNA expression by real-time PCR. Biotechniques.

