# Supplementary material for: Downregulation of lncRNA PpL-T31511 and Pp-miRn182 Promotes Hydrogen Cyanamide-Induced Endodormancy Release through the PP2C-H2O2 Pathway in Pear (Pyrus pyrifolia)
Source: Int J Mol Sci. 2021 Oct 31;22(21):11842. doi: 10.3390/ijms222111842 (PMC8584160; doi:10.3390/ijms222111842)
Supplement: Supplementary file 1 [file ijms-22-11842-s001.zip › Supplementary files/Figure S.pdf]

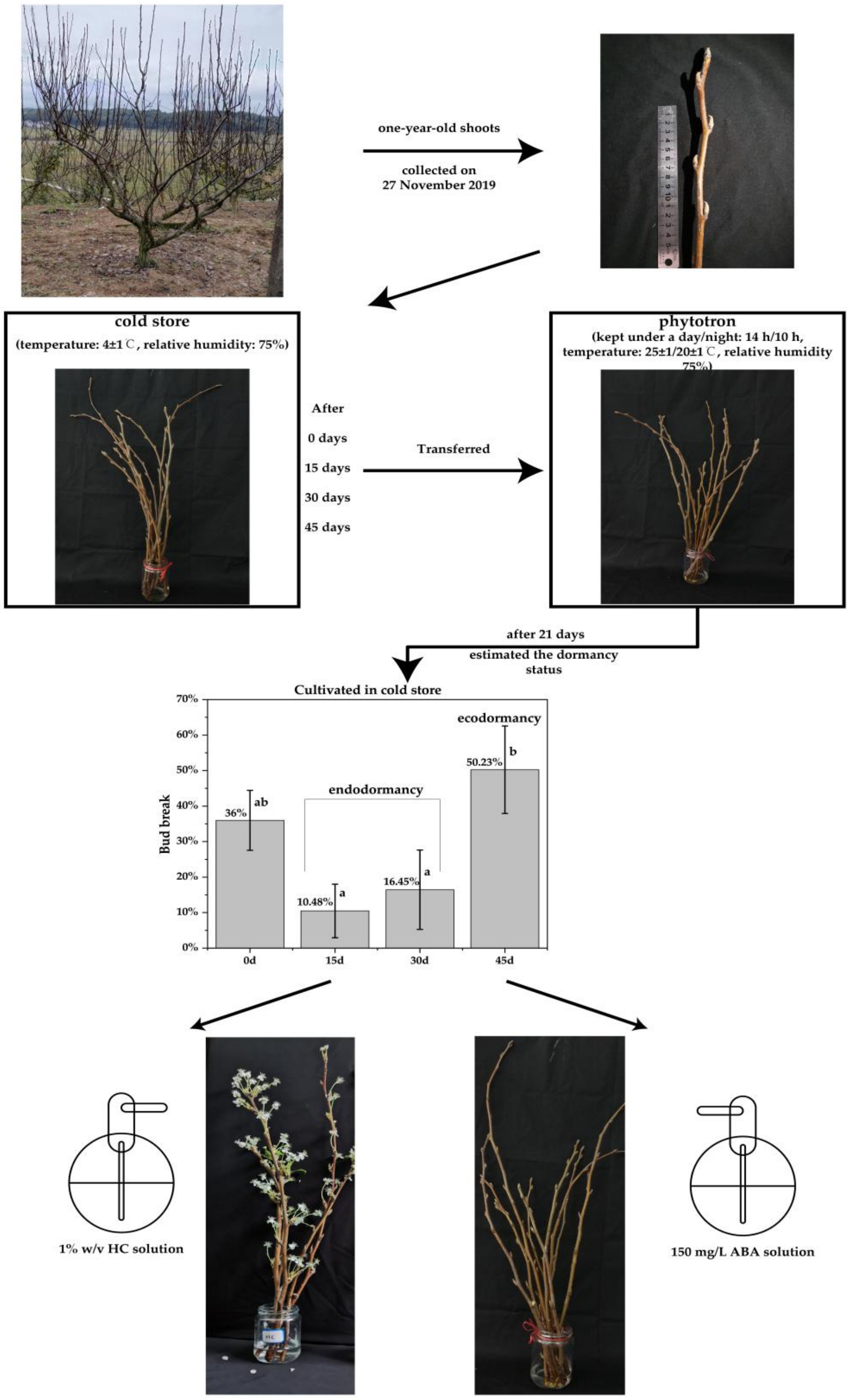

Figure S1. Experimental flow charts. One-year-old shoots (approximately 50-80 cm long and bearing 8-15 lateral floral buds) of *pyrus pyrifolia* '*Huanghua*' trees were collected on 27 November 2019. Then, the shoots were placed in water in vials in a cold store (temperature: 4 ±1℃, relative humidity: 75%). The water in the vials was changed every 7 days. After 15 days, 30 days and 45 days, the shoots were removed from cold store in batches and placed in a phytotron (kept under a day/night: 14 h/10 h, temperature: 25±1/20±1℃, relative humidity 75%) for estimating the dormancy status of lateral floral buds. Meanwhile, 1% w/v HC solution or water (defined as the H-CK group) was applied to endodormant floral buds (cultivated in cold store for 15 days). 150 mg/L ABA or water (defined as the A-CK group) was applied to ecodormant floral buds (cultivated in cold store for 45 days). After treatments, the shoots were placed in water in a phytotron. Lateral floral buds were collected from each treatments at 0 day, 0.5 days, 3 days and 9days after treatment. .

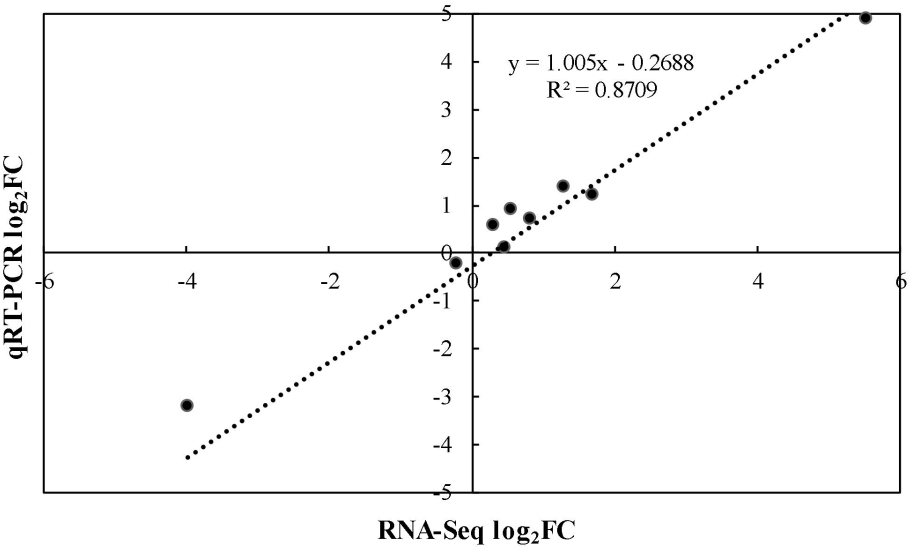

Figure S2. Coefficient analysis between gene expression ratios (log2FC) obtained from qRT-PCR and RNA-Seq data (compared with CK and C1 samples). **indicates a significant correlation at $p<0.01$.

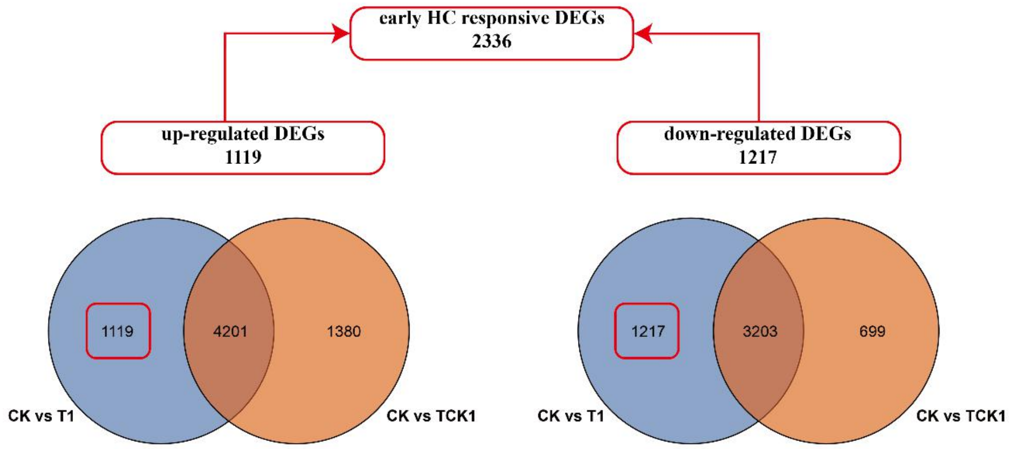

(**a**)

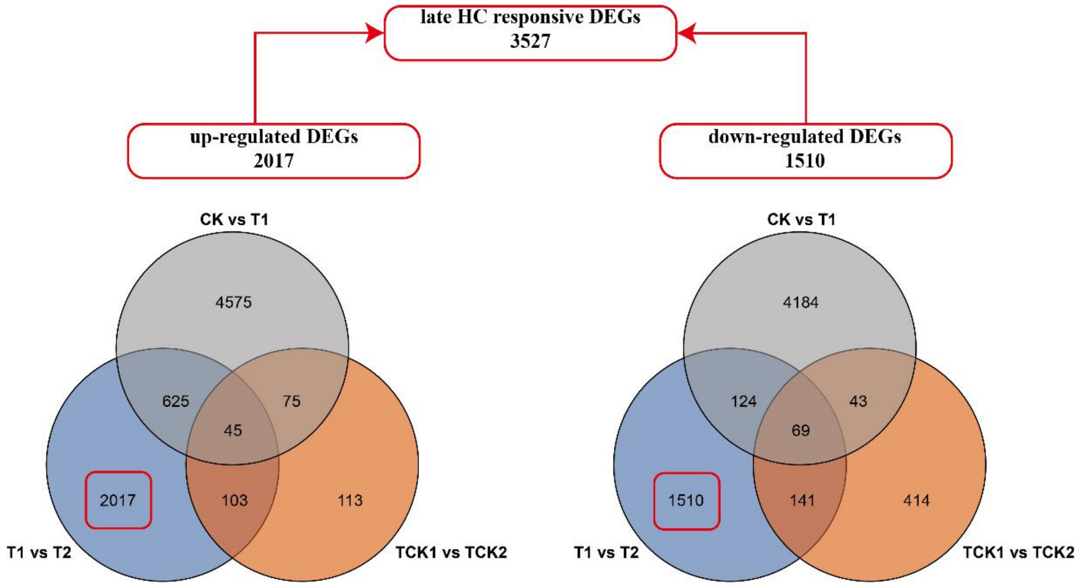

(**b**)

Figure S3. Venn diagrams of HC-regulated gens**. (a)** Venn diagrams of early HC responsive genes; **(b)** Venn diagrams of late HC responsive genes. The red box indicates the candidate DEGs.

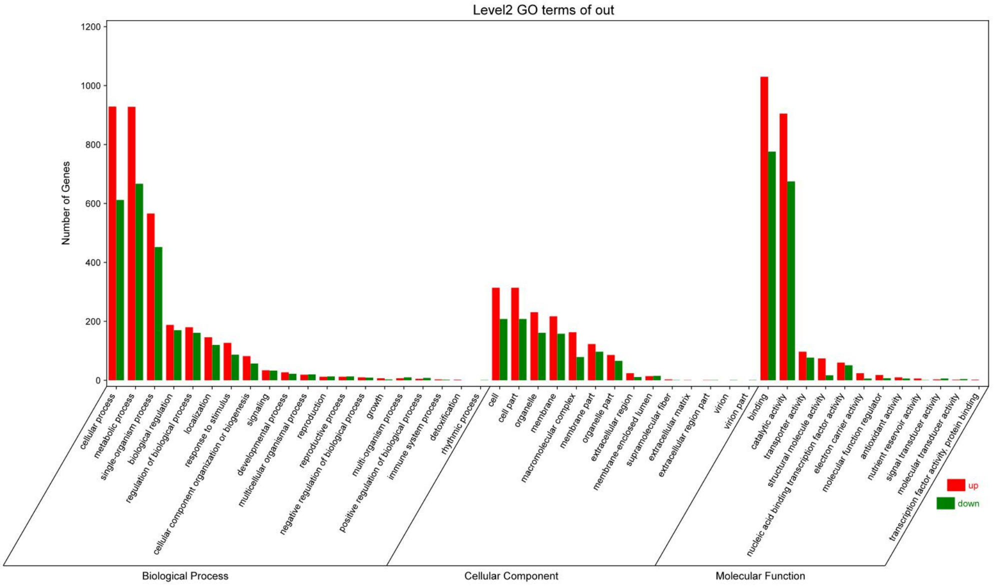

(**a**)

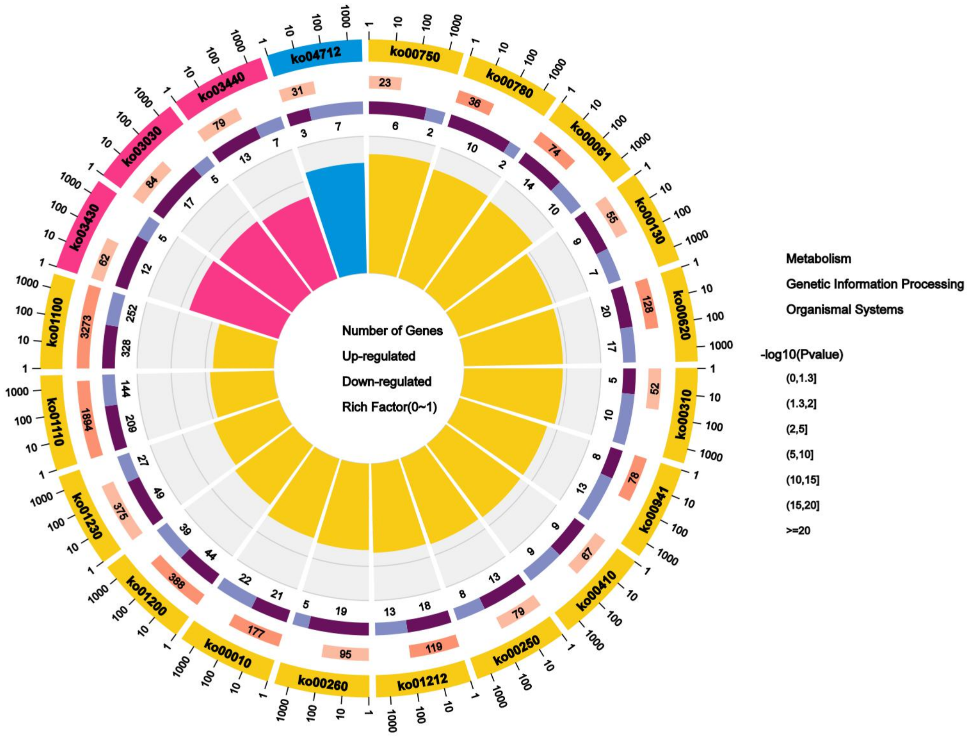

(**b**)

Figure S4. Venn diagrams of HC-regulated gens**. (a)** Gene Ontology enrichment analysis for DEGs in pear floral buds induced by HC treatment. The x-axis represents the second-order GO term, and y-axis represents the number of DEGs in this term; the red represents up- and the green represents down-regulation DEGs. **(b)** KEGG enrichment analysis for DEGs in pear floral buds induced by HC treatment. The first lap: pathways of top 20 enrichment, and different colors represents different KEGG A classes; the second lap: number and Q value of this pathway in background genes; the third lap: dark and light purple represents the proportion of up- and down-regulated genes, respectively; the innermost lap: RichFactor values of each pathway.

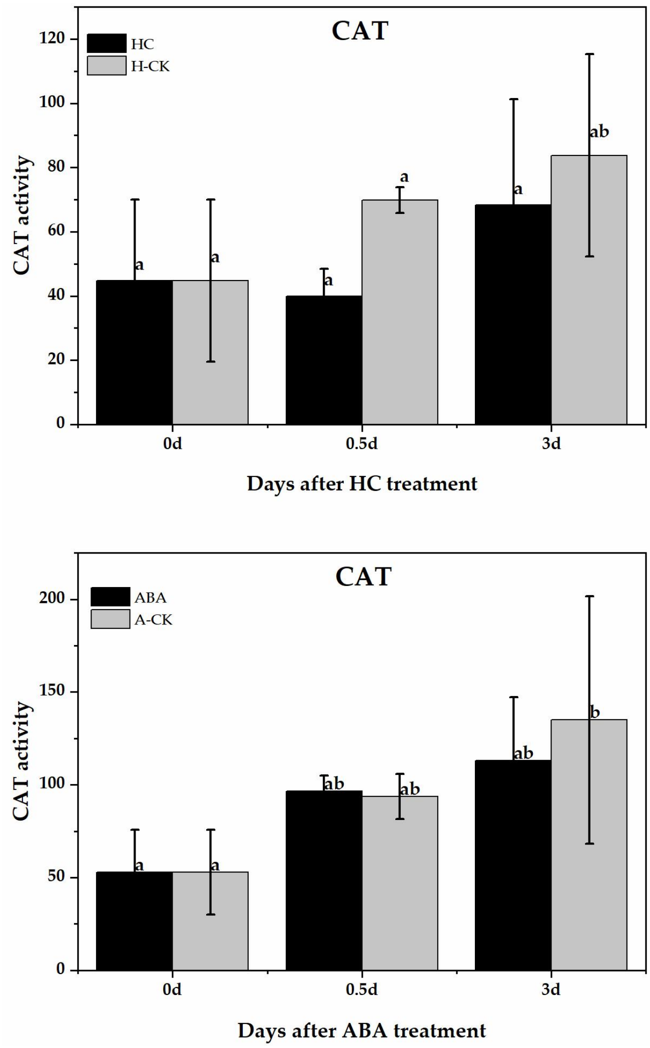

Figure S5. Changes in CAT activity after HC and ABA treatments in pear floral buds. Data are presented as the mean ± standard error of three biological replicates; different letters indicate significant differences ($P < 0.05$, Tukey's test) between samples.

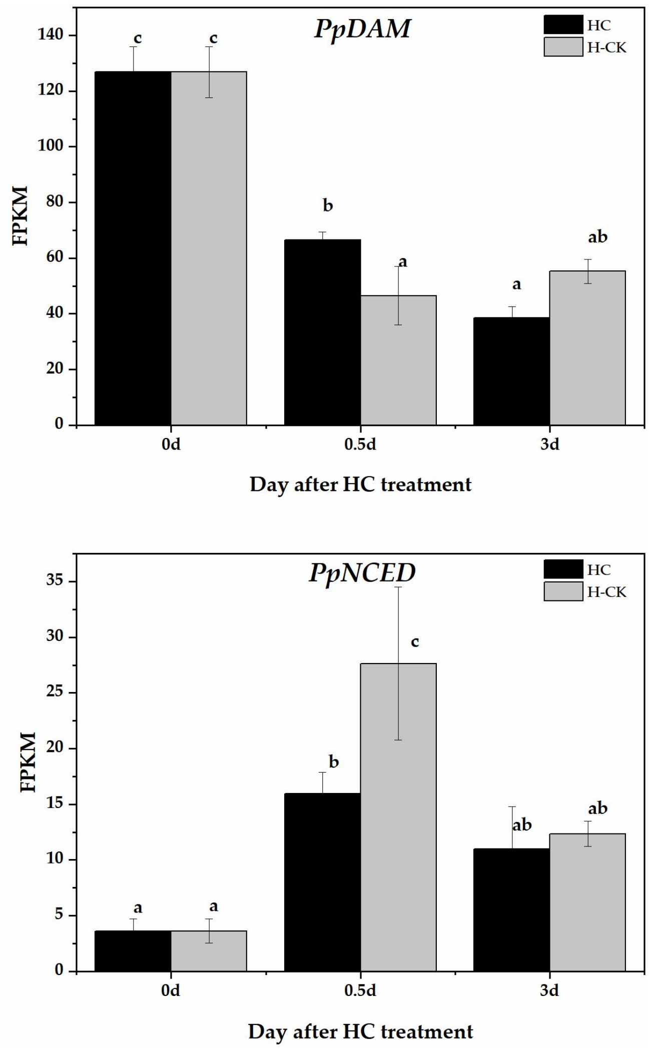

Figure S6. The relative expression of *PpDAM* and *PpNCED* in pear floral buds treated with HC. Data are presented as the mean ± standard error of three biological replicates; different letters indicate significant differences ($P < 0.05$, Tukey's test) between samples.

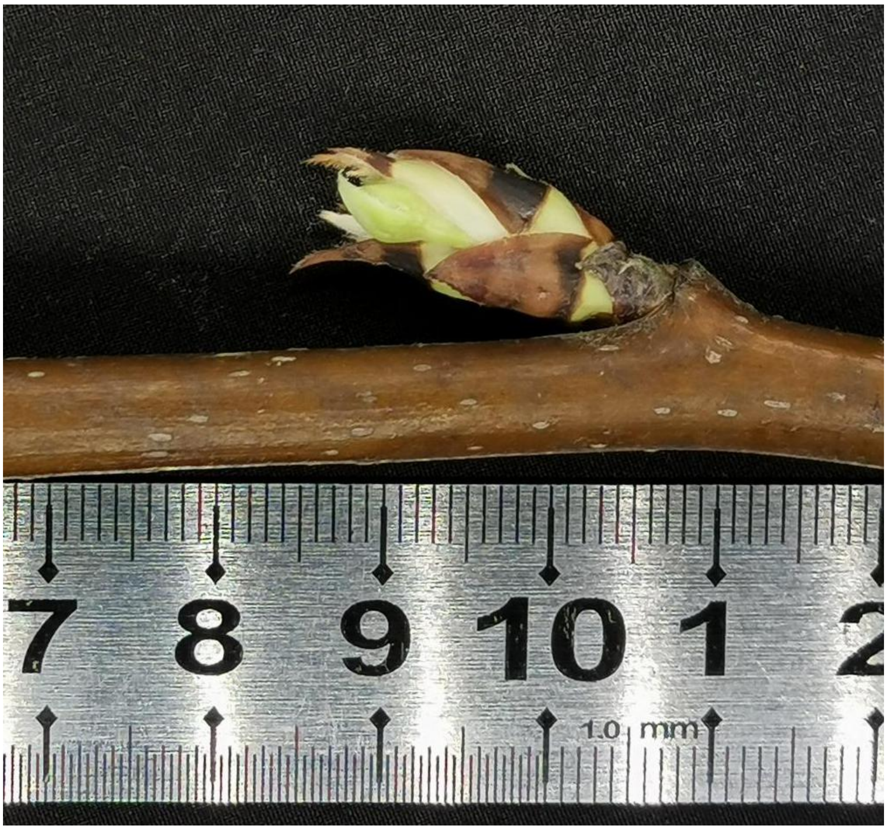

Figure S7. The photo of typical floral bud break. The beginning of bud break was defined as green leaf tips enclosing visible flowers.

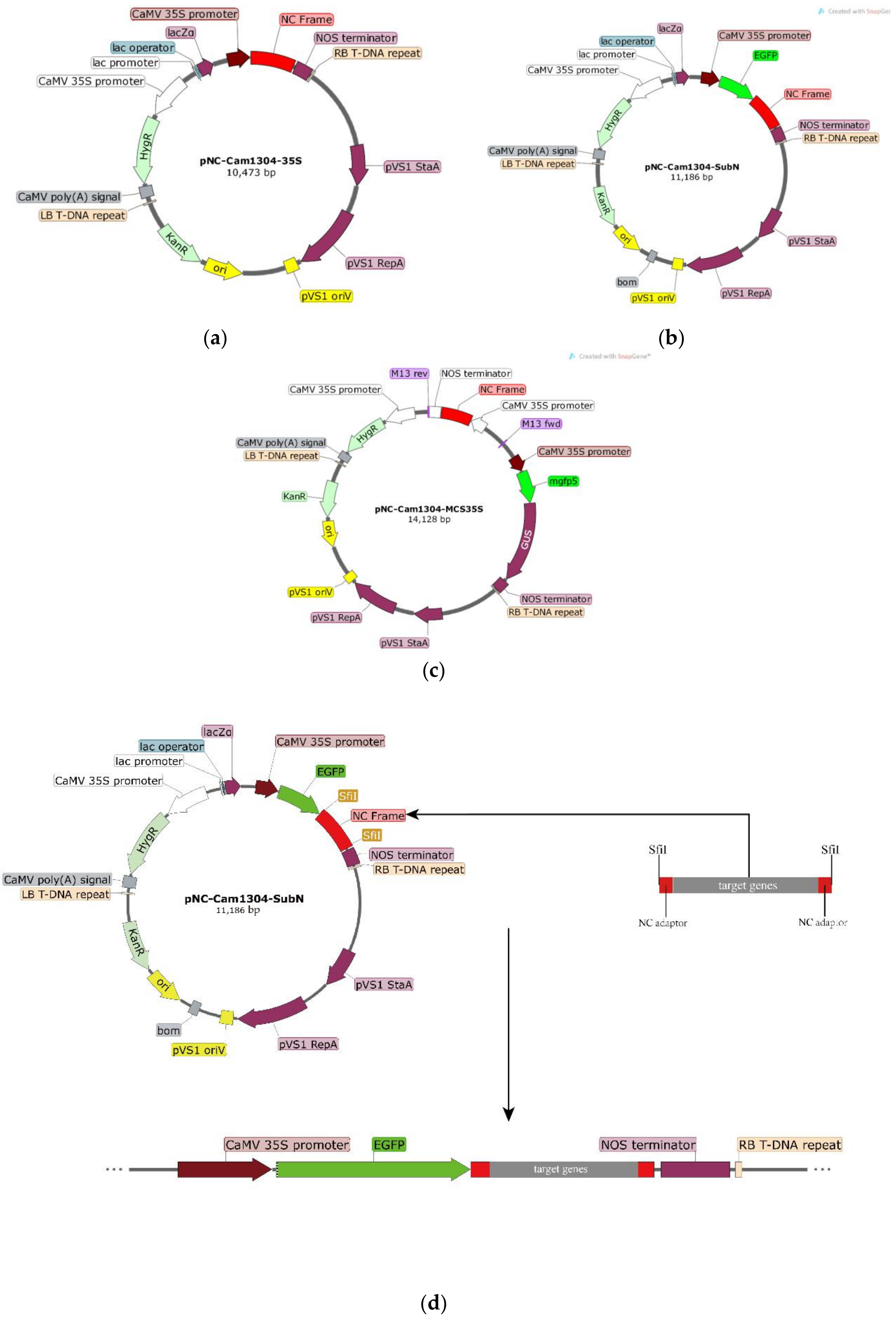

Figure S8. Structure diagram of vectors. **(a)** Structure diagram of pNC-Cam1304-35S vector. **(b)** Structure diagram of pNC-Cam1304-SubN vector. **(c)** Structure diagram of pNC-Cam1304-MCS35S vector. **(d)** Reconstruction diagram of pNC-Cam1304-SubN-target genes vector.
